# Impact of Hyperglycemia and Diabetes Mellitus on Breakthrough Mucormycosis Outcomes in Patients with Hematologic Malignancies—Complex and Intriguing Associations

**DOI:** 10.3390/jof9010045

**Published:** 2022-12-28

**Authors:** Alexander Franklin, Sebastian Wurster, Dierdre B. Axell-House, Ying Jiang, Dimitrios P. Kontoyiannis

**Affiliations:** 1 Department of Infectious Diseases, Infection Control and Employee Health, University of Texas MD Anderson Cancer Center, Houston, TX 77030, USA; 2 Section of Infectious Diseases, Department of Medicine, Baylor College of Medicine, Houston, TX 77030, USA; 3 Division of Infectious Diseases, Houston Methodist Hospital and Houston Methodist Research Institute, Houston, TX 77030, USA

**Keywords:** mucormycosis, hematologic malignancy, diabetes mellitus, hyperglycemia, insulin, mortality

## Abstract

Mucormycosis (MCR) is frequently associated with diabetic ketoacidosis and hyperglycemia, as well as hematologic malignancies (HMs) and hematopoietic stem cell transplantation (HSCT). However, little is known about the effect of hyperglycemia on MCR outcomes in patients with HMs. We therefore conducted a retrospective cohort study of adult patients hospitalized with MCR and HM or HSCT (*n* = 103) at MD Anderson Cancer Center from April 2000 through to April 2020. Twenty-three patients (22%) had documented episodes of severe hyperglycemia. Sixty patients had >5 serum glucose measurements within 28 days prior to MCR symptom onset; of those, 14 (23%) met the criteria for persistent hyperglycemia. Sixteen patients (16%) received insulin prior to admission. The crude mortality 42 days from the onset of MCR symptoms in our cohort was 31%. Neither severe nor persistent hyperglycemia were associated with excess mortality. Insulin use prior to index admission was associated with decreased 42-day mortality on univariate analysis (*p* = 0.031). In conclusion, in a setting of high crude mortality, severe and/or persistent hyperglycemia do not appear to be associated with excess mortality in patients with HM or HSCT developing MCR. Insulin use prior to MCR diagnosis may be associated with decreased mortality, although further research is needed to validate this effect and to study its mechanistic underpinnings.

## 1. Introduction

Invasive mucormycosis (MCR) is the third most common invasive fungal infection and second most common mold infection in patients with hematologic malignancies (HM) and those who have undergone hematopoietic stem cell transplantation (HSCT) [1,2]. Mortality from MCR in patients with HM and HSCT has been estimated to be as high as 48–66% [1,3] and can approach 100% for patients with central nervous system involvement or disseminated disease and those who do not recover from neutropenia [3,4].

Besides HMs, other predisposing risk factors for MCR include diabetes mellitus (DM), especially diabetic ketoacidosis, penetrating trauma, states of iron overload, treatment with the iron chelator deferoxamine or glucocorticosteroids (GCSs), and, recently, COVID-19 [2,5,6,7]. DM and hyperglycemia have also been shown to augment the risk for MCR in patients with other predisposing factors, such as COVID-19 [6,7].

Prior research has revealed differences in clinical presentation and outcomes of MCR depending on the underlying primary predisposing condition [2]. Specifically, patients whose primary risk factor is DM typically present with sino-orbital MCR and have more favorable prognosis compared to those with HM or HSCT who predominantly present with sino-pulmonary involvement or disseminated disease [8]. Although there is evidence that multiple predisposing risk factors can cumulatively result in adverse outcomes of MCR, little research exists exploring the specific interplay of both DM/hyperglycemia and HM or HSCT [9,10]. We therefore sought to further elucidate the specific effects of hyperglycemia and the receipt of antihyperglycemic therapies on outcomes of MCR in patients with underling HM or HSCT.

## 2. Materials and Methods

### 2.1. Study Design

This study was based on a previously curated retrospective cohort of patients with breakthrough MCR and HM or HSCT at the University of Texas MD Anderson Cancer Center, a tertiary oncologic center in Houston, Texas, between April 2000 and April 2020 [10]. Patients were included if they were ≥18 years old, met the European Organization for Research and Treatment of Cancer/National Institute of Allergy and Infectious Diseases Mycoses Study Group (EORTC/MSG) criteria for proven or probable MCR [11], and were on mold-active antifungal prophylaxis prior to diagnosis of MCR. 

### 2.2. Characteristics Assessed and Definitions

Previously recorded patient characteristics and treatment data included demographics (age, race/ethnicity, and sex), type and status of HM, HSCT status, presence of graft-versus-host disease (GvHD), immunosuppressive medications including GCSs, clinical presentation of MCR, absolute neutrophil count (ANC) and absolute lymphocyte count (ALC) at time of MCR diagnosis, mold-active prophylaxis and antifungal treatment regimens, and surgical treatments for MCR [10]. 

For the present study, we additionally reviewed data regarding hyperglycemia states and treatments, including history of DM, insulin and/or metformin prescription, fasting serum glucose level (sGL) closest to 28 days prior to MCR symptom onset, and fasting sGLs within 7 days prior to MCR symptom onset. We elected not to analyze data regarding hemoglobin A1C levels as these are thought to be less reliable in HM patients, many of whom are dependent on transfusion of blood products [12]. 

The presence of pancytopenia was recorded at the time of MCR diagnosis. Neutropenia and severe neutropenia were defined as absolute neutrophil count (ANC) ≤500 cells/µL and ≤100 cells/µL, respectively. Lymphopenia was defined as absolute lymphocyte count (ALC) ≤1000 cells/µL. Significant GCS use was defined as 600 mg prednisone or dose-equivalent received in the 30 days preceding diagnosis of MCR. Persistent hyperglycemia was defined as having both an average fasting sGL >126 mg/dL in the five most recent measurements prior to MCR symptom onset and a value of >126 mg/dL for the fasting sGL closest to 28 days prior to symptom onset. The level of 126 mg/dL was chosen based on American Diabetes Association definitions of DM [13]. Patients were excluded from this analysis if there was insufficient data on sGL to adjudicate persistent hyperglycemia. Severe hyperglycemia was defined as any recorded fasting sGL >180 mg/dL, including levels within 7 days of MCR symptom onset and the measurement closest to 28 days prior to MCR symptom onset. Diagnosis of known DM was based on the patients’ problem list and diagnoses in the electronic health record and/or in the most recent discharge note prior to index admission as well as the history and physical at time of index admission.

### 2.3. Outcomes and Statistical Analysis

The primary outcome was 42-day, all-cause mortality from the time of onset of MCR symptoms. Categorical variables were compared by chi-squared or Fisher’s exact test, as appropriate. Continuous variables were compared by Wilcoxon rank sum test. Univariate time-dependent survival analysis was performed for the main variables of interest (severe hyperglycemia, persistent hyperglycemia, and insulin use prior to index admission) using Kaplan–Meier curves and the Mantel–Cox log-rank test. Logistic regression analysis was used to identify independent predictors of 42-day mortality. Therefore, variables with a *p*-value of <0.20 on univariate analysis were included in an initial multivariate model that was then reduced to the final model by backward elimination. All tests were 2-sided with a significance level of 0.05. Data analysis and visualization were performed using Microsoft Office Excel 365 (Microsoft Corporation, Redmond, WA, USA), Prism v9 (GraphPad Software, San Diego, CA, USA), and SAS version 9.4 (SAS Institute Inc., Cary, NC, USA).

## 3. Results

### 3.1. Patients and Clinical Characteristics

All 103 patients with MCR in the previously reported database [10] had sGL measurements within 28 days from onset of MCR symptoms and were analyzed for the purpose of this study. Demographics and clinical characteristics of the 103 patients are summarized in Table 1. The median age was 52 (range 18–76), 67 patients (65%) were male, and 72 (70%) identified as non-Hispanic Caucasian. Leukemia and myelodysplastic syndrome (MDS) accounted for the majority of the malignancies (89%), with lymphoma and multiple myeloma accounting for the remaining 11%. The majority of patients (78%) had active cancer at the time of MCR diagnosis. About half of the patients (49%) had undergone allogenic HSCT. Forty out of the fifty patients (80%) who received allogenic HSCT experienced GvHD. Significant GCS use was common (35%), as was neutropenia at the time of MCR diagnosis (63%), with a median absolute neutrophil count of 40 at diagnosis (range 0–2660). Similarly, 91% of our patients had lymphopenia at the time of MCR diagnosis. The median time from MCR symptom onset to diagnosis (defined as the time of culture collection) was 7 days (IQR 4–14). The median Acute Physiologic Assessment and Chronic Health Evaluation II (APACHE II) score at MCR diagnosis was 14 (IQR 12–17) and 13% of the patients were in the intensive care unit (ICU) at the time of MCR diagnosis. The predominant clinical manifestation of MCR was sino-orbital infection (*n* = 38, 37%), whereas sino-pulmonary infection (*n* = 27, 26%), disseminated infection (*n* = 19, 18%), and other manifestations (*n* = 19, 18%) were less common in our cohort. 

### 3.2. Hyperglycemia Trends and Insulin Therapy

The median fasting sGL closest to MCR symptom onset was 125 (IQR 105–150), and the median fasting sGL 28 days prior to symptoms onset was 117 (IQR 99–147). Twenty-three patients (22%) met the criteria for severe hyperglycemia with at least one fasting sGL of >180. Of the patients with sufficient sGL measurements for analysis (*n* = 60), 14 (23%) met the criteria for persistent hyperglycemia. Sixteen patients (16%) received insulin prior to index admission, and 12 (12%) were on metformin prior to index admission. There was no significant difference in terms of persistent hyperglycemia between patients with DM who were prescribed insulin prior to admission (75%) and diabetic patients not on insulin (50%, *p* = 0.47). Although diabetic patients on insulin tended to have a higher likelihood of severe hyperglycemia (71%) than those on oral antidiabetics or on no anti-diabetic pharmacotherapy (21%), this trend did not reach significance (*p* = 0.07).

### 3.3. Outcomes

Thirty-two out of the one hundred and three patients died within 42 days of MCR symptom onset (31% all-cause mortality). As previously published [10], active malignancy (91% versus 72%, *p* = 0.03), unrecovered neutropenia (53% versus 10%, *p* < 0.001), prior prophylactic use of Mucorales-active antifungals (31% versus 8% *p* < 0.01), and treatment in the ICU at the time of MCR diagnosis (34% versus 3%, *p* < 0.001) were associated with significantly increased 42-day mortality from the time of the symptom onset of MCR on univariate analysis. Furthermore, higher APACHE II scores at the time of MCR diagnosis (median, 19 versus 13, *p* < 0.001) and a lack of surgical MCR treatment (19% versus 58%, *p* < 0.001) were associated with increased 42-day mortality. Moreover, (sino-)pulmonary MCR was more common in non-survivors than in survivors (44% versus 18%), whereas 64% of the survivors had isolated sinusitis, sino-orbital MCR, or other localized extrapulmonary manifestations (*p* = 0.04).

**Figure 1:**
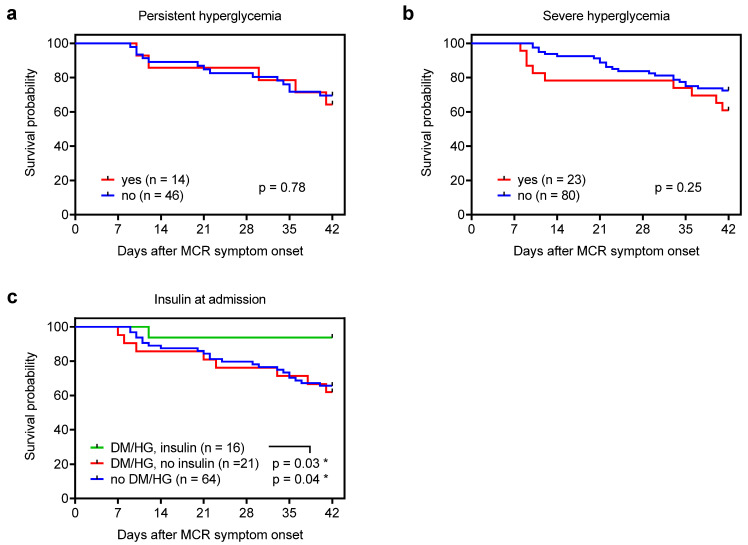
Survival probability depending on (**a**) persistent hyperglycemia, (**b**) presence of severe hyperglycemia, and (**c**) presence of diabetes mellitus (DM)/hyperglycemia (HG) and insulin treatment at admission. Data regarding persistent hyperglycemia and insulin use were available for 60 and 101 out of the 103 patients, respectively. Mantel–Cox log-rank test. MCR = mucormycosis.

Interestingly, neither persistent nor severe hyperglycemia were associated with 42-day, all-cause mortality on univariate analysis (Table 1, Figure 1a,b). Insulin use, however, was associated with decreased 42-day, all-cause mortality on univariate analysis (Table 1, *p* = 0.02). Furthermore, insulin use was associated with better mortality outcomes on time-dependent 42-day survival analysis compared to both patients without DM/hyperglycemia (*p* = 0.04) and those with DM/hyperglycemia not receiving insulin (*p* = 0.03, Figure 1c).

Factors independently associated with 42-day, all-cause mortality on multivariate logistic regression analysis were neutropenia status (OR 0.35, *p* = 0.03 for neutropenia with recovery, and OR 5.33, *p* < 0.001 for neutropenia without recovery; reference: non-neutropenic patients), ICU admission at time of MCR diagnosis (OR 4.98, *p* < 0.001), Mucorales-active antifungal prophylaxis prior to diagnosis (OR 3.21, *p* < 0.01), and lymphoma/myeloma as underlying malignancy (OR 3.21, *p* = 0.02) (Table 2). None of the hyperglycemia trends and antidiabetic treatments reviewed in the present study, i.e., persistent hyperglycemia, severe hyperglycemia, or insulin prescription, were independent predictors of 42-day mortality.

## 4. Discussion

Studies in patients with acute leukemia and transplant recipients have shown that hyperglycemia is a significant risk factor for infections, including invasive mold infections [14,15,16]. Consequently, strict glucose control has been shown to lead to fewer overall infections after HSCT [14]. In addition, pathogenesis studies and clinical data identified hyperglycemia as a well-established risk factor for the development of MCR in the non-HM setting [8,17]. However, there is a paucity of data regarding the impact of hyperglycemia on MCR in patients with HM and/or HSCT. To our knowledge, this is the first large cohort of patients with MCR in the setting of HM to report in detail on hyperglycemic trends and DM treatments.

There is increasing evidence illustrating complex immune–metabolic interactions in diabetic patients [18]. Specifically, impaired cellular immunity, enhanced fungal iron acquisition, more favorable fungal growth conditions, and upregulated expression of cellular entry targets for Mucorales are thought to contribute to hyperglycemia-associated susceptibility to MCR [5]. For instance, studies in diabetic mouse models have shown that DM leads to impaired phagocytosis, impaired chemotaxis, and decreased myeloperoxidase activity in neutrophils [19,20]. In fact, even transient hyperglycemia, lasting for as little as eight hours, can hamper human neutrophil degranulation [21]. In addition, DM has also been shown to have pleiotropic deleterious effects on macrophage signaling pathways, which may play a role in suboptimal immune response [22]. Lastly, hyperglycemia has been associated with lower rates and duration of complete remission in patients with HM, which, in turn, is a strong predictor of the risk and severity of invasive mold infections [15,23,24].

However, we herein found that, in a cohort of HM/HSCT patients where neutropenia was seen in almost two thirds of patients (severe neutropenia in over half of patients) and nearly one in four had disseminated MCR (Table 1) and in the setting of high crude mortality, neither severe nor persistent hyperglycemia were associated with excess mortality. Similarly, in an independent prospective cohort of primarily neutropenic patients with HM and MCR who had high mortality (DEFEAT study), DM also did not correlate with outcomes [25]. In addition to the low number of MCR cases with which to study such a complex question, there are other potential explanations for the absence of an association between hyperglycemia and mortality in our cohort. First, patients with HM and HSCT have multiple ”competing” causes of death. This is reflected by the fact that ICU stay at diagnosis was independently associated with the increased 42-day mortality of MCR. Similarly, many of the patients in our cohort had multiple (combined or sequential) and profound insults to their cellular immunity, including dysfunctional immune defense due to an active underlying malignancy, HSCT, and its treatment. In this setting, a dissection of the impact of hyperglycemia on the overall net state of immunosuppression is difficult. Furthermore, our study was restricted to patients with proven or probable MCR. By definition, these patients have histopathologic or cytopathologic evidence of fungal infection, or culture growth. This suggests that these patients may have had a higher fungal burden than those with possible MCR [11]. An examination of patients who are diagnosed earlier, such as those identified via Mucorales PCR [26], may identify a sub-population in which the effects of hyperglycemia are more pronounced and its control more impactful. This question should be addressed in future research.

The lack of impact of hyperglycemia on outcomes in our cohort of highly immunocompromised patients, who had a high background risk for mortality, contrasts with the influence of the correction of hyperglycemia and ketoacidosis on MCR in experimental models [27] and in non-HM patients with DM, a condition with a lower risk of death [8,9,28]. In fact, spontaneous resolution of MCR without antifungal therapy has been rarely described in the context of the rapid correction of the metabolic disorder [29]. Finally, whether GCSs, which have a pleiotropic deleterious effect in fungal immunity [30], independently influence the prognostic significance of hyperglycemia remains a subject of future investigations.

Interestingly, we found that insulin use at or before the index admission was associated with the decreased 42-day mortality of MCR on univariate analysis and time-dependent survival analysis; however, this was not confirmed as an independent predictor of mortality on multivariate analysis. There has been experimental evidence that insulin use might have an epigenetic effect on metabolic reprogramming in the broader context of “nutritional immunity” against various classes of pathogens [18,31]. For instance, in vitro studies support a role for insulin in enhancing macrophage-mediated immunity. Specifically, the deletion of genes encoding for molecules involved in insulin signaling in macrophages led to an anti-inflammatory phenotype, with decreased secretion of pro-inflammatory factors upon stimulation [32]. Furthermore, insulin receptor signaling can enhance the expression of major histocompatibility complex molecules that are required for optimal antigen presentation to T cells, polarize host immunity toward an adaptive lymphoid cell-driven response, and boost T-cell immunity through nutrient uptake and enhancement of glycolytic capacity [33]. However, the specific effects of insulin and insulin receptor signaling on anti-Mucoralean host immunity are unknown and remain to be studied in the future.

Our study had several limitations related to its retrospective nature and its low power. Although the diagnosis of MCR has remained unchanged throughout the study period, this cohort spanned twenty years. Therefore, there has been significant heterogeneity in terms of oncologic and antifungal treatment approaches and supportive care protocols. Additionally, all-cause, 42-day mortality was used instead of MCR-attributable mortality. This was a practical decision based on the difficulty of arbitrating cause of death in a retrospective cohort, given the very low autopsy rates in contemporary patients with HM [34] and the many competing factors that may contribute to mortality. Additionally, we did not assess mortality outcomes in hyperglycemic patients depending on the successful correction of hyperglycemia during MCR treatment (e.g., fasting sGL consistently below 126 mg/dl during the first week after MCR diagnosis). Finally, while this is one of the larger published cohorts of MCR in patients with HM or HSCT, cases still remain limited, and large multicenter cohorts or meta-analysis combining multiple prior cohorts might be better equipped to confirm an effect of insulin use on mortality in this population.

## 5. Conclusions

In a setting of high crude mortality and neutropenia, severe and persistent hyperglycemia do not appear to be associated with excess mortality in patients with HMs or HSCT developing MCR. However, insulin use prior to MCR diagnosis may be associated with decreased 42-day mortality. In view of emerging in vitro and animal studies supporting a salutary immunomodulatory role for insulin signaling, further validation of this potential effect of insulin on MCR mortality is needed by clinical and experimental studies.

## Figures and Tables

**Table 1:** Comparison of patients with MCR depending on 42-day outcomes from symptom onset.

	42-Day Outcome			
Characteristics	Total (*n* = 103)	Survived (*n* = 71)	Died (*n* = 32)	*p*-Value
Age (years), median (range)	52 (18–76)	52 (18–76)	54 (23–75)	0.24
Sex, male, *n* (%)	67 (65)	46 (65)	21 (66)	0.93
Race, *n* (%)				0.85
White	72 (70)	50 (70)	22 (69)	
Black	11 (11)	7 (10)	4 (13)	
Asian	6 (6)	5 (7)	1 (3)	
Hispanic	14 (14)	9 (13)	5 (16)	
Type of cancer, *n* (%)				**0.03**
Leukemia/myelodysplastic syndrome	92 (89)	67 (94)	25 (78)	
Lymphoma/multiple myeloma	11 (11)	4 (6)	7 (22)	
Active malignancy, *n* (%)	80 (78)	51 (72)	29 (91)	**0.03**
Allogenic HSCT, *n* (%)	50 (49)	38 (54)	12 (38)	0.13
GvHD, *n* (% among allogenic HSCT recipients)	40/50 (80)	30/38 (79)	10/12 (83)	>0.99
Significant GCS use, *n* (%)	36 (35)	22 (31)	14 (44)	0.21
ANC at diagnosis, median (IQR)	40 (0–2660)	240 (0–3560)	0 (0–790)	0.08
Neutropenia (ANC ≤ 500/µL) status, *n* (%) Non-neutropenic Neutropenia, recovered Neutropenia, not recovered	38 (37) 41 (40) 24 (23)	29 (41) 35 (49) 7 (10)	9 (28) 6 (19) 17 (53)	**<0.001**
Severe neutropenia (ANC ≤ 100/µL), *n* (%)	56 (54)	35 (49)	21 (66)	0.12
ALC at diagnosis, median (IQR)	120 (0–530)	200 (0–550)	45 (0–400)	0.25
Lymphocytopenia (ALC ≤ 1000), *n* (%)	94 (91)	66 (93)	28 (88)	0.45
DM or GCS-induced hyperglycemia, *n* (%)	37 (36)	28 (39)	9 (28)	0.27
GCS-induced hyperglycemia, *n* (%)	19 (18)	16 (23)	3 (9)	0.11
DM (not GCS-induced), *n* (%)	18 (17)	12 (17)	6 (19)	0.82
DM with insulin use, *n* (%)	7 (7)	6 (8)	1 (3)	0.43
DM without insulin, *n* (%)	11 (11)	6 (8)	5 (16)	0.31
Insulin at admission for MCR, *n* (%) ^a^	16/101 (16)	15/70 (21)	1/31 (3)	**0.02**
Metformin at admission for MCR, *n* (%) ^b^	12/102 (12)	8/70 (11)	4 (13)	>0.99
Persistent hyperglycemia, *n* (%)	14/60 (23)	9/41 (22)	5/19 (26)	0.75
Severe hyperglycemia, *n* (%)	23 (22)	14 (20)	9 (28)	0.34
Mucor-active antifungal prophylaxis, *n* (%)	16 (16)	6 (8)	10 (31)	**<0.01**
Time (days) from MCR symptom onset to diagnosis (culture collection), median (IQR)	7 (4–14)	9 (5–16)	6 (4–11)	**0.03**
APACHE II score at MCR diagnosis, median (IQR)	14 (12–17)	13 (11–16)	19 (14–20)	**<0.001**
ICU at time of MCR diagnosis, *n* (%)	13 (13)	2 (3)	11 (34)	**<0.001**
Clinical presentation of MCR, *n* (%) Sinusitis/sino-orbital Pneumonia/sino-pulmonary infection Disseminated infection Other manifestations	38 (37) 27 (26) 19 (18) 19 (18) ^c^	29 (41) 13 (18) 13 (18) 16 (23) ^d^	9 (28) 14 (44) 6 (19) 3 (9) ^e^	**0.04**
Surgical treatment of MCR, *n* (%)	47 (46)	41 (58)	6 (19)	**<0.001**
Antifungal therapy of MCR, *n* (%) Monotherapy Combination therapy	17 (17) ^f^ 86 (83) ^i^	13 (18) ^g^ 58 (82) ^j^	4 (13) ^h^ 28 (87) ^k^	0.46

Percentages use each column’s cohort size as denominator (unless indicated otherwise, i.e., if a variable only refers to a sub-cohort). ^a^ unknown in 2 patients; ^b^ unknown in 1 patient; ^c^ 12 skin/soft tissue, 3 gastro-intestinal, 2 liver, 2 mandibular abscess; ^d^ 9 skin/soft tissue, 3 gastro-intestinal, 2 liver, 2 mandibular abscess; ^e^ 3 skin/soft tissue; ^f^ 15 AMB, 2 POSA; ^g^ 11 AMB, 2 POSA; ^h^ 4 AMB; ^i^ 26 AMB + POSA, 21 AMB + CAS, 31 AMB + CAS + ISA, 8 AMB + ISA; ^j^ 19 AMB + POSA, 8 AMB + CAS, 26 AMB + CAS + ISA, 5 AMB + ISA; ^k^ 7 AMB + POSA, 13 AMB + CAS, 5 AMB + CAS + ISA, 3 AMB + ISA. Abbreviations: ALC = absolute lymphocyte count, ANC = absolute neutrophil count, APACHE II = Acute Physiologic Assessment and Chronic Health Evaluation II, CAS = caspofungin, DM = diabetes mellitus, GvHD = graft-versus-host disease, HSCT = hematopoietic stem cell transplant, ICU = intensive care unit, ISA = isavuconazole, LAmB = liposomal amphotericin B, MCR = mucormycosis, POSA = posaconazole. Bold font indicates *p* value ≤ 0.05.

**Table 2:** Multivariable logistic regression model of predictors of 42-day mortality since MCR symptom onset.

Independent Predictor	Adjusted OR	95% CI	*p*-Value
Neutropenia Status No neutropenia at diagnosis Neutropenia, recovered Neutropenia, not recovered	Reference 0.35 5.33	0.11 to 0.91 1.85 to 18.75	**0.03** **<0.001**
ICU at diagnosis	4.98	1.83 to 18.16	**<0.001**
Mucorales-active antifungal prophylaxis	3.07	1.32 to 7.86	**<0.01**
Type of underlying malignancy Leukemia/myelodysplastic syndrome Lymphoma/multiple myeloma	Reference 3.21	1.18 to 9.25	**0.02**

Abbreviations: CI = confidence interval, ICU = intensive care unit, OR = odds ratio. Bold font indicates *p* value ≤ 0.05.

## Data Availability

Not applicable.
